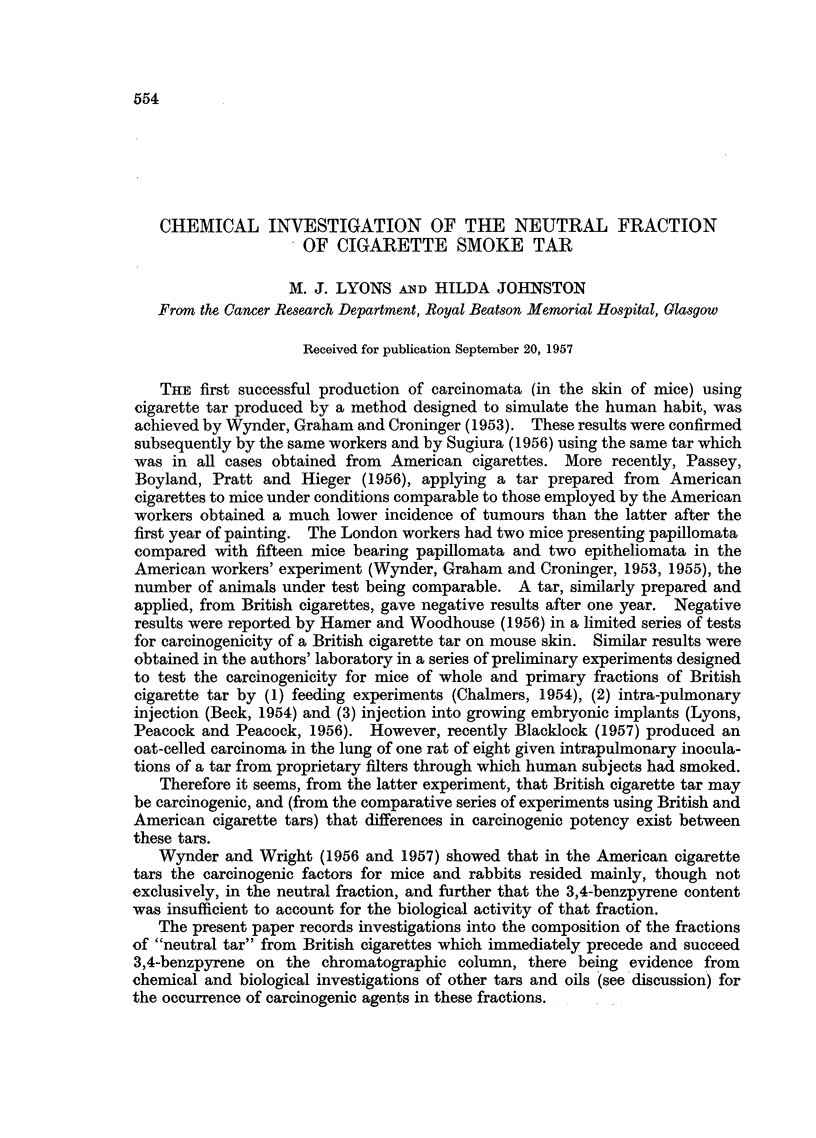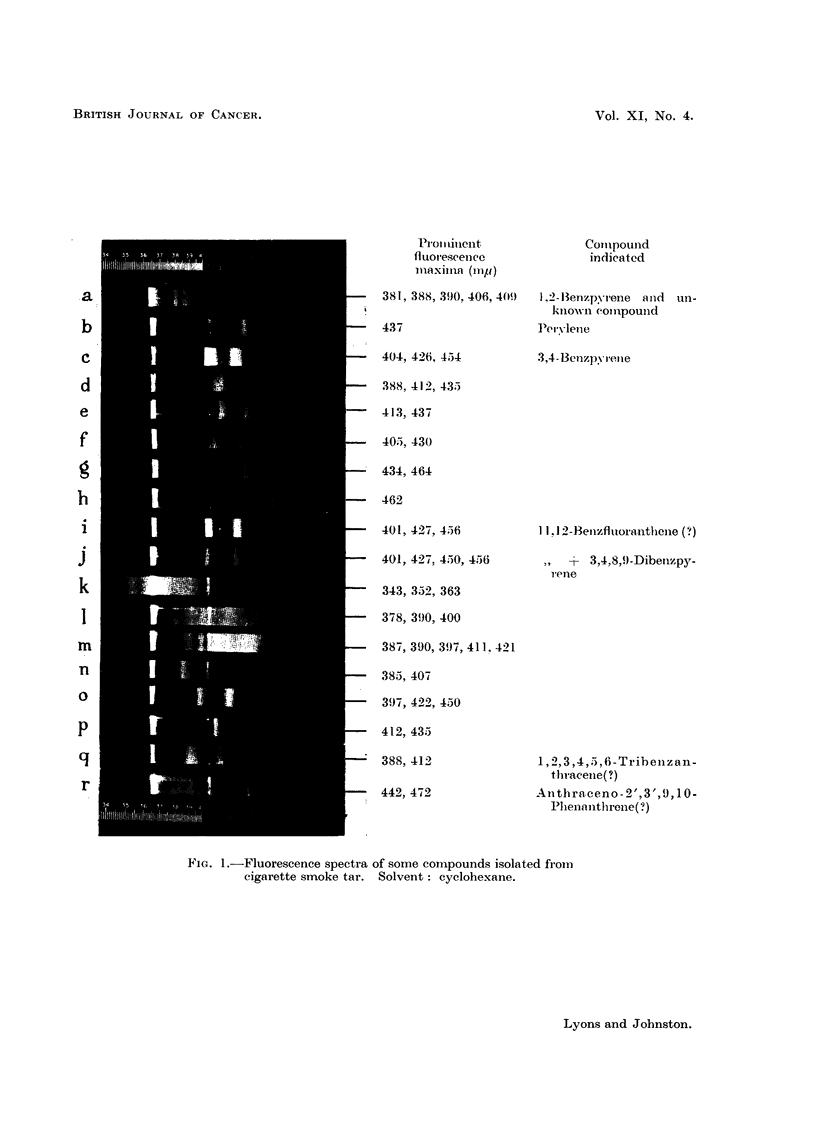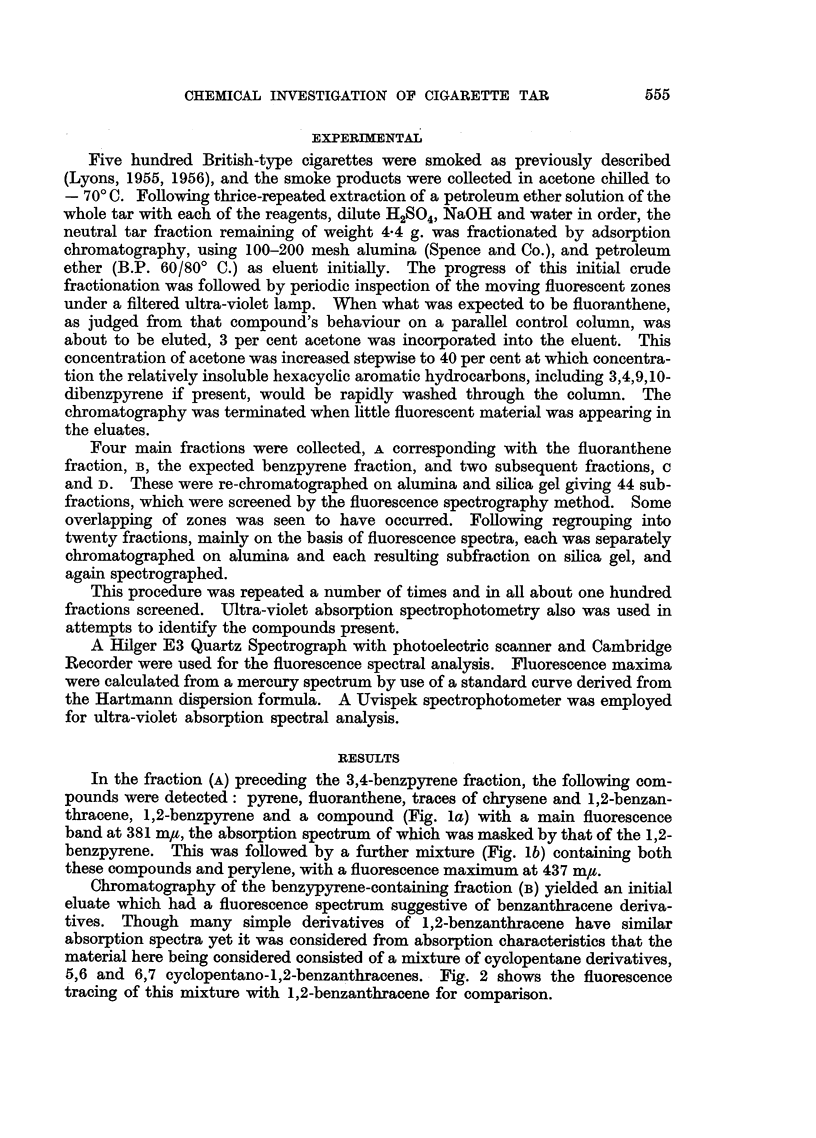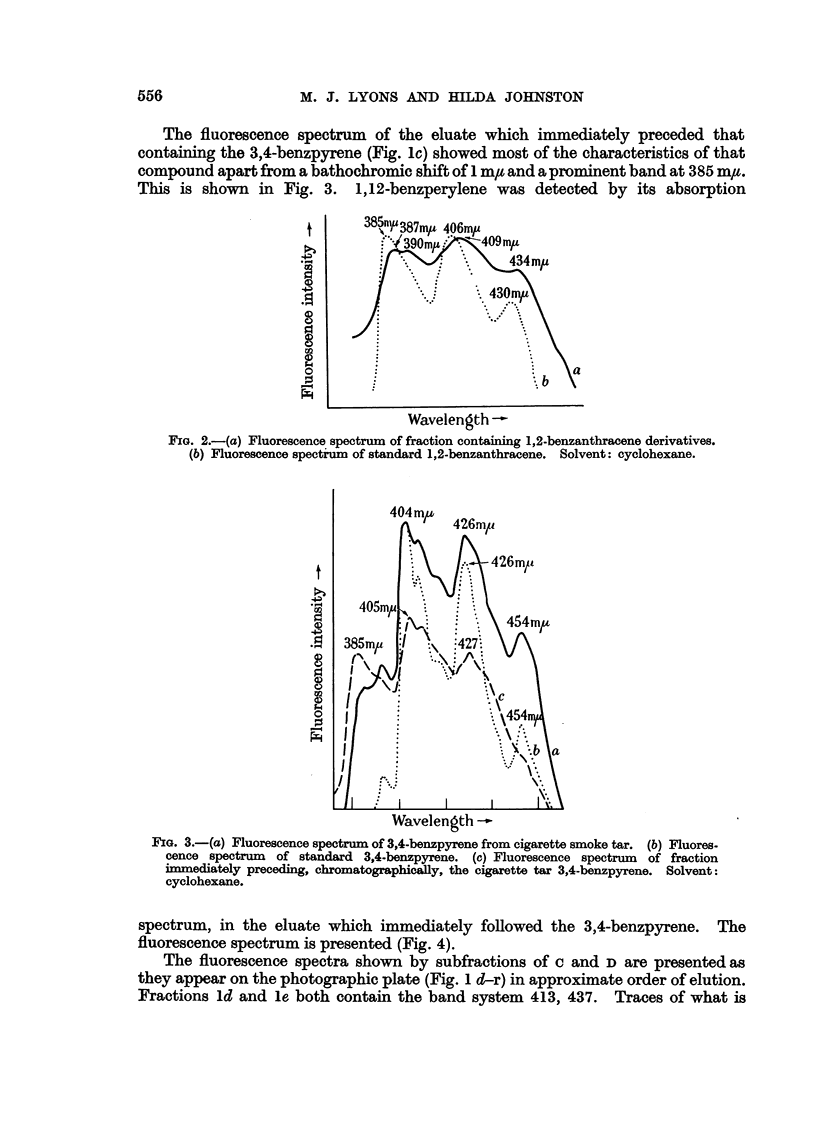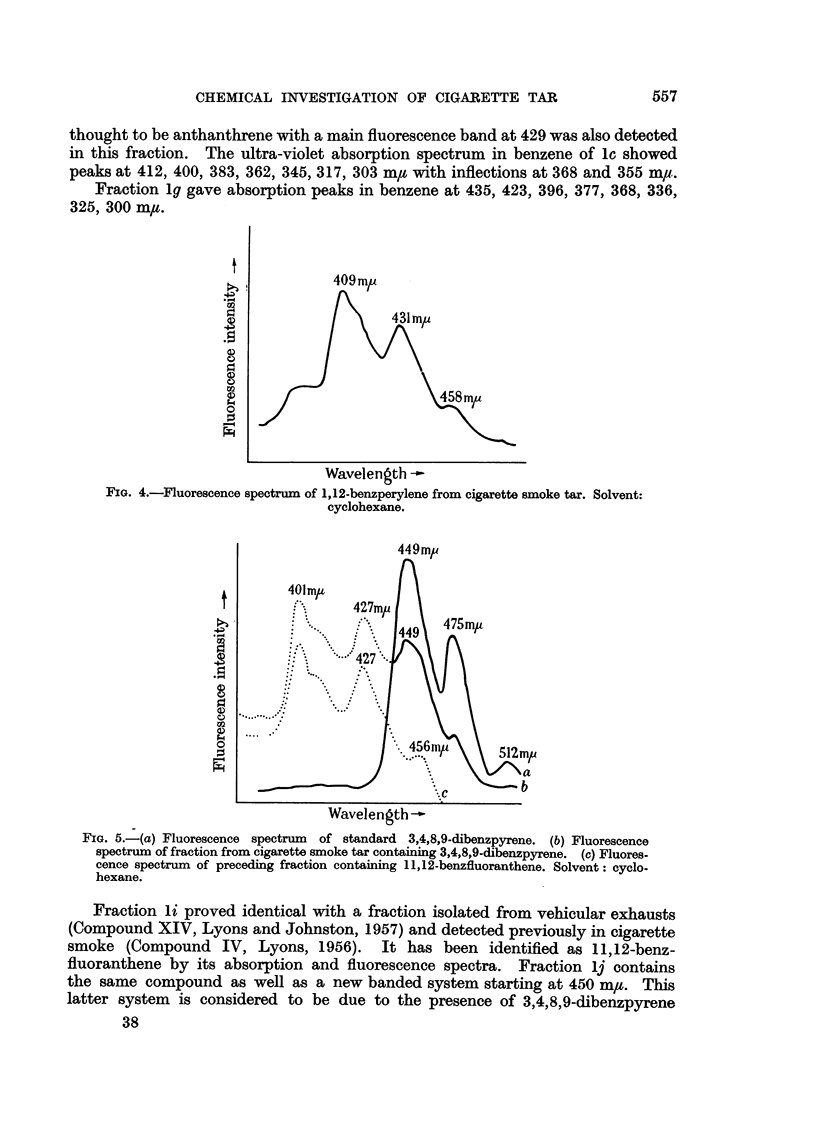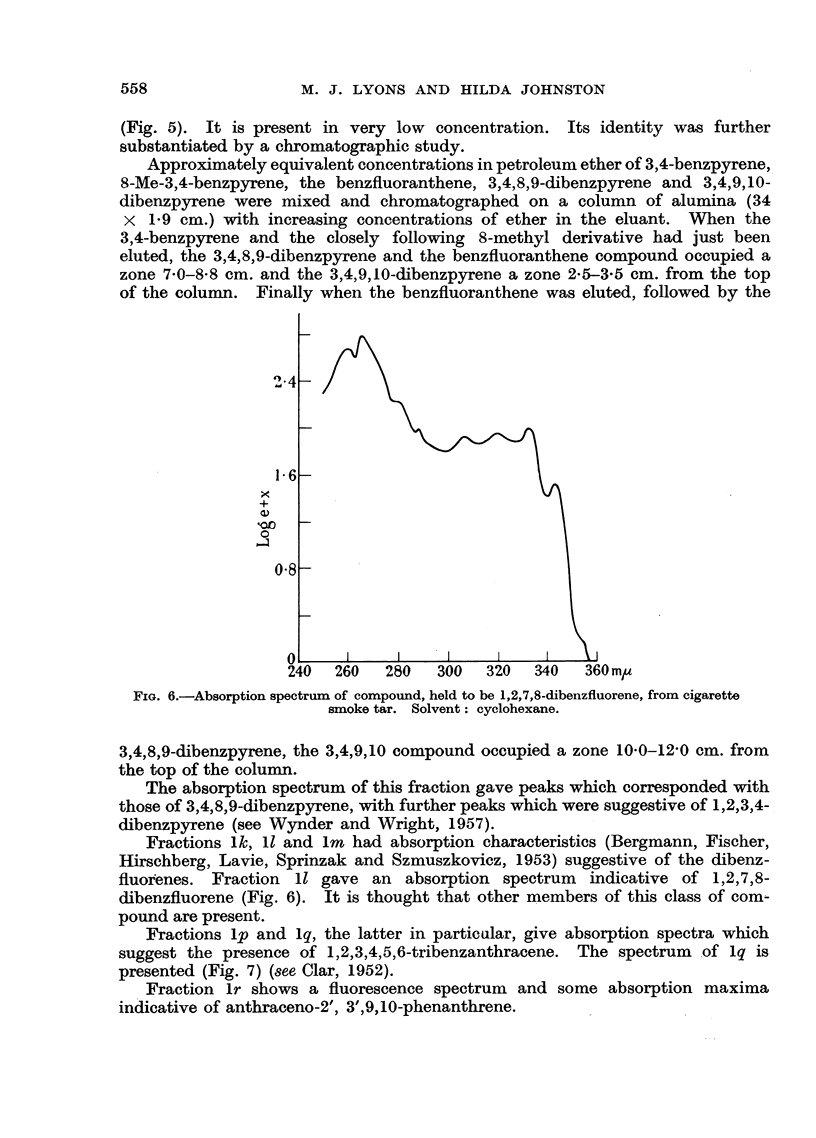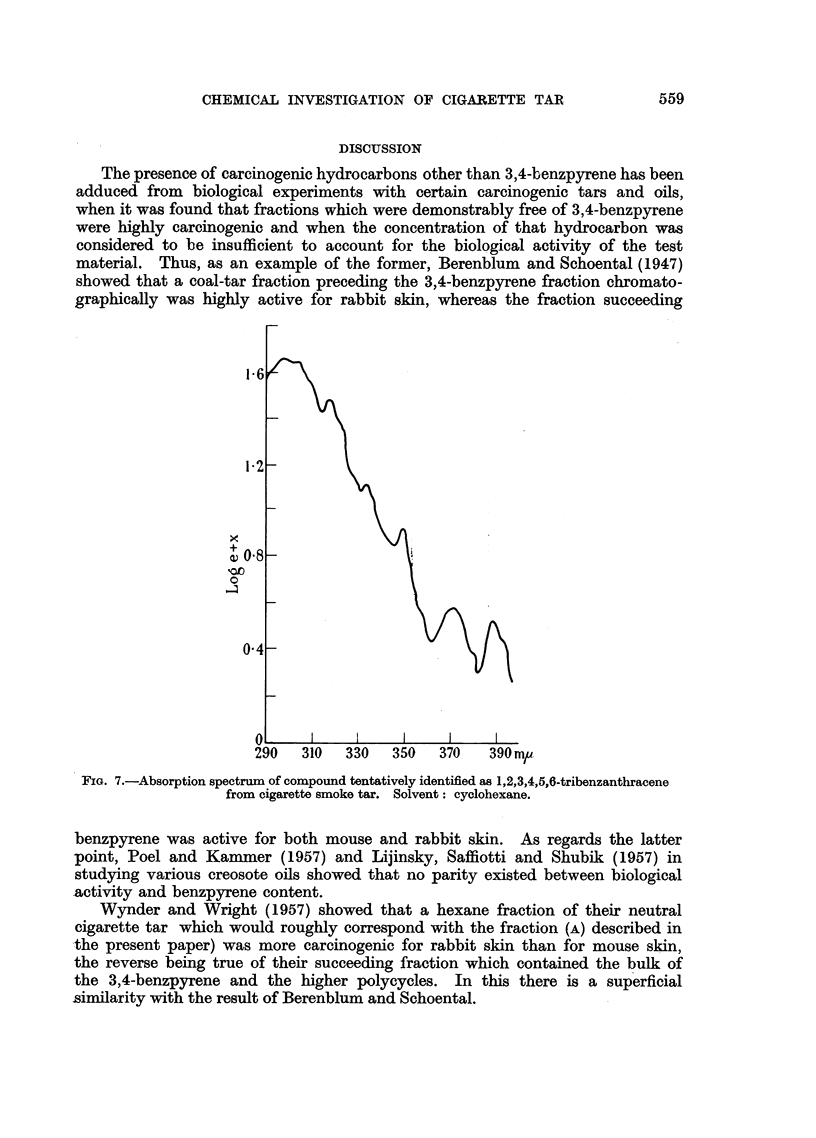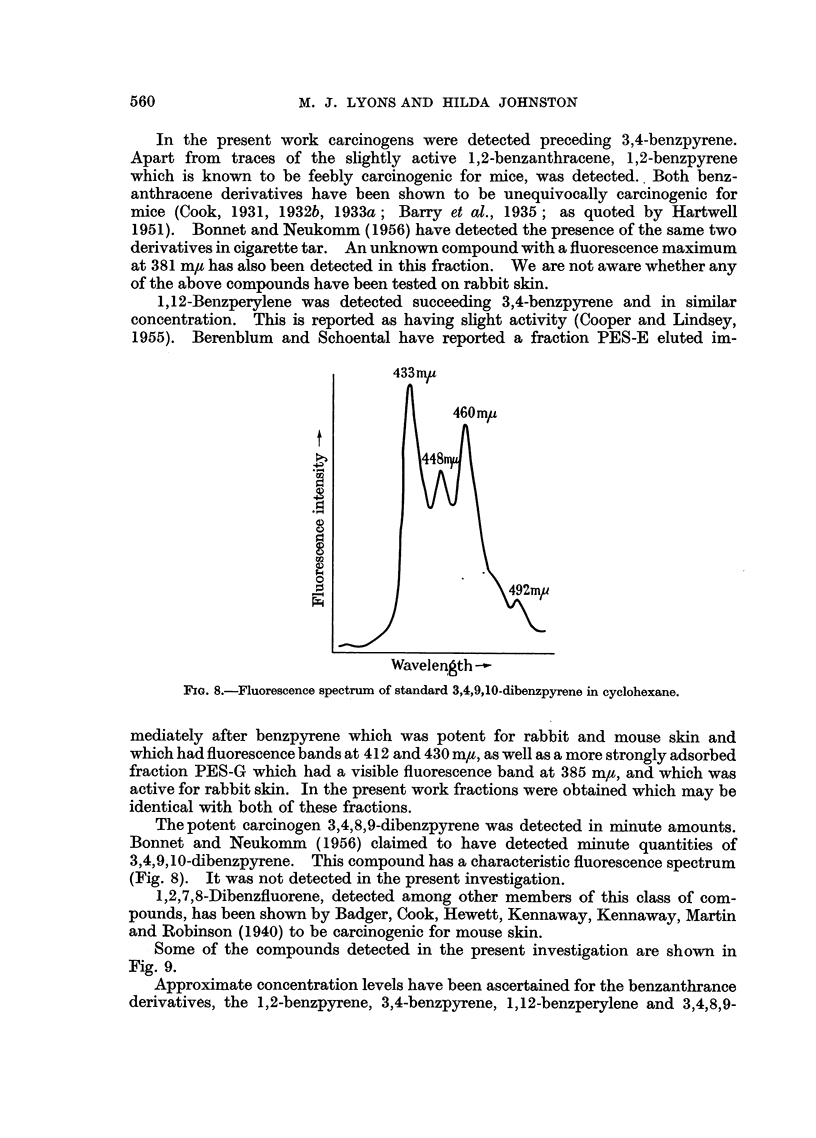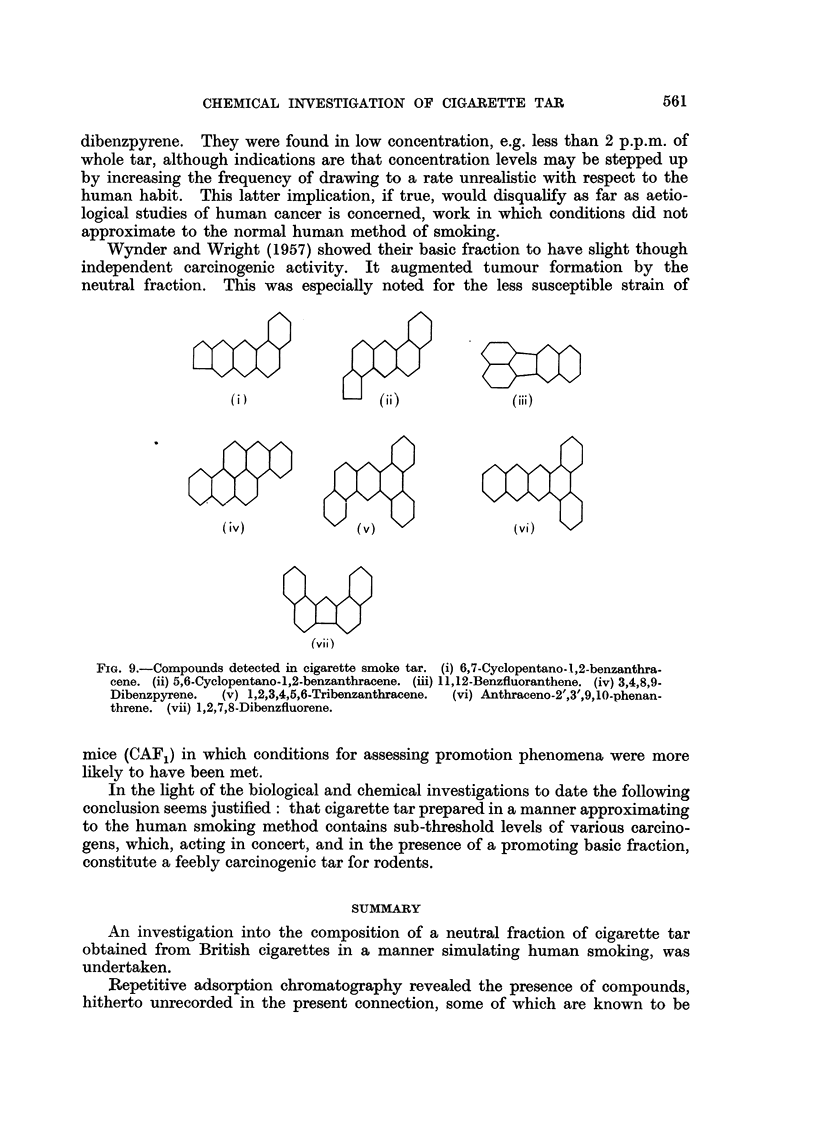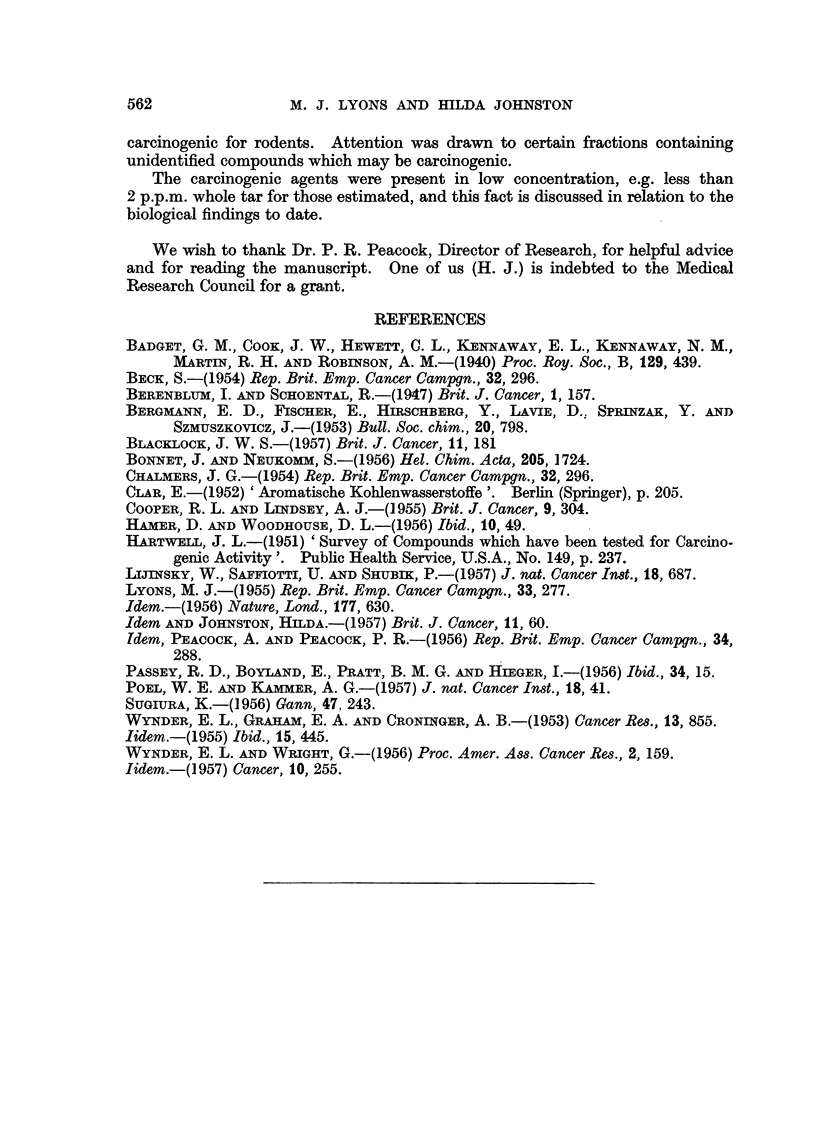# Chemical Investigation of the Neutral Fraction of Cigarette Smoke Tar

**DOI:** 10.1038/bjc.1957.68

**Published:** 1957-12

**Authors:** M. J. Lyons, Hilda Johnston

## Abstract

**Images:**


### CHEMICAL INVESTIGATION OF THE NEUTRAL FRACTION OF CIGARETTE SMOKE TAR

M. J. LYONS AND HILDA JOHNSTON

*From the Cancer Research Department, Royal Beatson Memorial Hospital, Glasgow*

Received for publication September 20, 1957

THE first successful production of carcinomata (in the skin of mice) using cigarette tar produced by a method designed to simulate the human habit, was achieved by Wynder, Graham and Croninger (1953). These results were confirmed subsequently by the same workers and by Sugiura (1956) using the same tar which was in all cases obtained from American cigarettes. More recently, Passey, Boyland, Pratt and Hieger (1956), applying a tar prepared from American cigarettes to mice under conditions comparable to those employed by the American workers obtained a much lower incidence of tumours than the latter after the first year of painting. The London workers had two mice presenting papillomata compared with fifteen mice bearing papillomata and two epitheliomata in the American workers' experiment (Wynder, Graham and Croninger, 1953, 1955), the number of animals under test being comparable. A tar, similarly prepared and applied, from British cigarettes, gave negative results after one year. Negative results were reported by Hamer and Woodhouse (1956) in a limited series of tests for carcinogenicity of a British cigarette tar on mouse skin. Similar results were obtained in the authors' laboratory in a series of preliminary experiments designed to test the carcinogenicity for mice of whole and primary fractions of British cigarette tar by (1) feeding experiments (Chalmers, 1954), (2) intra-pulmonary injection (Beck, 1954) and (3) injection into growing embryonic implants (Lyons, Peacock and Peacock, 1956). However, recently Blacklock (1957) produced an oat-celled carcinoma in the lung of one rat of eight given intrapulmonary inoculations of a tar from proprietary filters through which human subjects had smoked.

Therefore it seems, from the latter experiment, that British cigarette tar may be carcinogenic, and (from the comparative series of experiments using British and American cigarette tars) that differences in carcinogenic potency exist between these tars.

Wynder and Wright (1956 and 1957) showed that in the American cigarette tars the carcinogenic factors for mice and rabbits resided mainly, though not exclusively, in the neutral fraction, and further that the 3,4-benzpyrene content was insufficient to account for the biological activity of that fraction.

The present paper records investigations into the composition of the fractions of "neutral tar" from British cigarettes which immediately precede and succeed 3,4-benzpyrene on the chromatographic column, there being evidence from chemical and biological investigations of other tars and oils (see discussion) for the occurrence of carcinogenic agents in these fractions.

| | Prominent fluorescence maxima (mμ) | Compound indicated |
|---|---|---|
| a | 381, 388, 390, 406, 409 | 1,2-Benzpyrene and unknown compound |
| b | 437 | Perylene |
| c | 404, 426, 454 | 3,4-Benzpyrene |
| d | 388, 412, 435 | |
| e | 413, 437 | |
| f | 405, 430 | |
| g | 434, 464 | |
| h | 462 | |
| i | 401, 427, 456 | 11,12-Benzfluoranthene (?) |
| j | 401, 427, 450, 456 | ,, + 3,4,8,9-Dibenzpyrene |
| k | 343, 352, 363 | |
| l | 378, 390, 400 | |
| m | 387, 390, 397, 411, 421 | |
| n | 385, 407 | |
| o | 397, 422, 450 | |
| p | 412, 435 | |
| q | 388, 412 | 1,2,3,4,5,6-Tribenzanthracene(?) |
| r | 442, 472 | Anthraceno-2′,3′,9,10-Phenanthrene(?) |

FIG. 1.—Fluorescence spectra of some compounds isolated from cigarette smoke tar. Solvent : cyclohexane.

### EXPERIMENTAL

Five hundred British-type cigarettes were smoked as previously described (Lyons, 1955, 1956), and the smoke products were collected in acetone chilled to — 70° C. Following thrice-repeated extraction of a petroleum ether solution of the whole tar with each of the reagents, dilute $H_2SO_4$, NaOH and water in order, the neutral tar fraction remaining of weight 4·4 g. was fractionated by adsorption chromatography, using 100–200 mesh alumina (Spence and Co.), and petroleum ether (B.P. 60/80° C.) as eluent initially. The progress of this initial crude fractionation was followed by periodic inspection of the moving fluorescent zones under a filtered ultra-violet lamp. When what was expected to be fluoranthene, as judged from that compound's behaviour on a parallel control column, was about to be eluted, 3 per cent acetone was incorporated into the eluent. This concentration of acetone was increased stepwise to 40 per cent at which concentration the relatively insoluble hexacyclic aromatic hydrocarbons, including 3,4,9,10-dibenzpyrene if present, would be rapidly washed through the column. The chromatography was terminated when little fluorescent material was appearing in the eluates.

Four main fractions were collected, A corresponding with the fluoranthene fraction, B, the expected benzpyrene fraction, and two subsequent fractions, C and D. These were re-chromatographed on alumina and silica gel giving 44 subfractions, which were screened by the fluorescence spectrography method. Some overlapping of zones was seen to have occurred. Following regrouping into twenty fractions, mainly on the basis of fluorescence spectra, each was separately chromatographed on alumina and each resulting subfraction on silica gel, and again spectrographed.

This procedure was repeated a number of times and in all about one hundred fractions screened. Ultra-violet absorption spectrophotometry also was used in attempts to identify the compounds present.

A Hilger E3 Quartz Spectrograph with photoelectric scanner and Cambridge Recorder were used for the fluorescence spectral analysis. Fluorescence maxima were calculated from a mercury spectrum by use of a standard curve derived from the Hartmann dispersion formula. A Uvispek spectrophotometer was employed for ultra-violet absorption spectral analysis.

### RESULTS

In the fraction (A) preceding the 3,4-benzpyrene fraction, the following compounds were detected : pyrene, fluoranthene, traces of chrysene and 1,2-benzanthracene, 1,2-benzpyrene and a compound (Fig. 1*a*) with a main fluorescence band at 381 m$\mu$, the absorption spectrum of which was masked by that of the 1,2-benzpyrene. This was followed by a further mixture (Fig. 1*b*) containing both these compounds and perylene, with a fluorescence maximum at 437 m$\mu$.

Chromatography of the benzypyrene-containing fraction (B) yielded an initial eluate which had a fluorescence spectrum suggestive of benzanthracene derivatives. Though many simple derivatives of 1,2-benzanthracene have similar absorption spectra yet it was considered from absorption characteristics that the material here being considered consisted of a mixture of cyclopentane derivatives, 5,6 and 6,7 cyclopentano-1,2-benzanthracenes. Fig. 2 shows the fluorescence tracing of this mixture with 1,2-benzanthracene for comparison.

The fluorescence spectrum of the eluate which immediately preceded that containing the 3,4-benzpyrene (Fig. 1*c*) showed most of the characteristics of that compound apart from a bathochromic shift of 1 mμ and a prominent band at 385 mμ. This is shown in Fig. 3. 1,12-benzperylene was detected by its absorption

FIG. 2.—(*a*) Fluorescence spectrum of fraction containing 1,2-benzanthracene derivatives. (*b*) Fluorescence spectrum of standard 1,2-benzanthracene. Solvent: cyclohexane.

FIG. 3.—(*a*) Fluorescence spectrum of 3,4-benzpyrene from cigarette smoke tar. (*b*) Fluorescence spectrum of standard 3,4-benzpyrene. (*c*) Fluorescence spectrum of fraction immediately preceding, chromatographically, the cigarette tar 3,4-benzpyrene. Solvent: cyclohexane.

spectrum, in the eluate which immediately followed the 3,4-benzpyrene. The fluorescence spectrum is presented (Fig. 4).

The fluorescence spectra shown by subfractions of C and D are presented as they appear on the photographic plate (Fig. 1 *d*–r) in approximate order of elution. Fractions 1*d* and 1*e* both contain the band system 413, 437. Traces of what is thought to be anthanthrene with a main fluorescence band at 429 was also detected in this fraction. The ultra-violet absorption spectrum in benzene of 1*c* showed peaks at 412, 400, 383, 362, 345, 317, 303 mμ with inflections at 368 and 355 mμ.

Fraction 1*g* gave absorption peaks in benzene at 435, 423, 396, 377, 368, 336, 325, 300 mμ.

Fig. 4.—Fluorescence spectrum of 1,12-benzperylene from cigarette smoke tar. Solvent: cyclohexane.

Fig. 5.—(*a*) Fluorescence spectrum of standard 3,4,8,9-dibenzpyrene. (*b*) Fluorescence spectrum of fraction from cigarette smoke tar containing 3,4,8,9-dibenzpyrene. (*c*) Fluorescence spectrum of preceding fraction containing 11,12-benzfluoranthene. Solvent: cyclohexane.

Fraction 1*i* proved identical with a fraction isolated from vehicular exhausts (Compound XIV, Lyons and Johnston, 1957) and detected previously in cigarette smoke (Compound IV, Lyons, 1956). It has been identified as 11,12-benzfluoranthene by its absorption and fluorescence spectra. Fraction 1*j* contains the same compound as well as a new banded system starting at 450 mμ. This latter system is considered to be due to the presence of 3,4,8,9-dibenzpyrene

(Fig. 5). It is present in very low concentration. Its identity was further substantiated by a chromatographic study.

Approximately equivalent concentrations in petroleum ether of 3,4-benzpyrene, 8-Me-3,4-benzpyrene, the benzfluoranthene, 3,4,8,9-dibenzpyrene and 3,4,9,10-dibenzpyrene were mixed and chromatographed on a column of alumina (34 × 1·9 cm.) with increasing concentrations of ether in the eluant. When the 3,4-benzpyrene and the closely following 8-methyl derivative had just been eluted, the 3,4,8,9-dibenzpyrene and the benzfluoranthene compound occupied a zone 7·0–8·8 cm. and the 3,4,9,10-dibenzpyrene a zone 2·5–3·5 cm. from the top of the column. Finally when the benzfluoranthene was eluted, followed by the

FIG. 6.—Absorption spectrum of compound, held to be 1,2,7,8-dibenzfluorene, from cigarette smoke tar. Solvent : cyclohexane.

3,4,8,9-dibenzpyrene, the 3,4,9,10 compound occupied a zone 10·0–12·0 cm. from the top of the column.

The absorption spectrum of this fraction gave peaks which corresponded with those of 3,4,8,9-dibenzpyrene, with further peaks which were suggestive of 1,2,3,4-dibenzpyrene (see Wynder and Wright, 1957).

Fractions 1*k*, 1*l* and 1*m* had absorption characteristics (Bergmann, Fischer, Hirschberg, Lavie, Sprinzak and Szmuszkovicz, 1953) suggestive of the dibenzfluorenes. Fraction 1*l* gave an absorption spectrum indicative of 1,2,7,8-dibenzfluorene (Fig. 6). It is thought that other members of this class of compound are present.

Fractions 1*p* and 1*q*, the latter in particular, give absorption spectra which suggest the presence of 1,2,3,4,5,6-tribenzanthracene. The spectrum of 1*q* is presented (Fig. 7) (*see* Clar, 1952).

Fraction 1*r* shows a fluorescence spectrum and some absorption maxima indicative of anthraceno-2′, 3′,9,10-phenanthrene.

DISCUSSION

The presence of carcinogenic hydrocarbons other than 3,4-benzpyrene has been adduced from biological experiments with certain carcinogenic tars and oils, when it was found that fractions which were demonstrably free of 3,4-benzpyrene were highly carcinogenic and when the concentration of that hydrocarbon was considered to be insufficient to account for the biological activity of the test material. Thus, as an example of the former, Berenblum and Schoental (1947) showed that a coal-tar fraction preceding the 3,4-benzpyrene fraction chromatographically was highly active for rabbit skin, whereas the fraction succeeding

FIG. 7.—Absorption spectrum of compound tentatively identified as 1,2,3,4,5,6-tribenzanthracene from cigarette smoke tar. Solvent : cyclohexane.

benzpyrene was active for both mouse and rabbit skin. As regards the latter point, Poel and Kammer (1957) and Lijinsky, Saffiotti and Shubik (1957) in studying various creosote oils showed that no parity existed between biological activity and benzpyrene content.

Wynder and Wright (1957) showed that a hexane fraction of their neutral cigarette tar which would roughly correspond with the fraction (A) described in the present paper) was more carcinogenic for rabbit skin than for mouse skin, the reverse being true of their succeeding fraction which contained the bulk of the 3,4-benzpyrene and the higher polycycles. In this there is a superficial similarity with the result of Berenblum and Schoental.

In the present work carcinogens were detected preceding 3,4-benzpyrene. Apart from traces of the slightly active 1,2-benzanthracene, 1,2-benzpyrene which is known to be feebly carcinogenic for mice, was detected. Both benzanthracene derivatives have been shown to be unequivocally carcinogenic for mice (Cook, 1931, 1932*b*, 1933*a*; Barry *et al.*, 1935; as quoted by Hartwell 1951). Bonnet and Neukomm (1956) have detected the presence of the same two derivatives in cigarette tar. An unknown compound with a fluorescence maximum at 381 m$\mu$ has also been detected in this fraction. We are not aware whether any of the above compounds have been tested on rabbit skin.

1,12-Benzperylene was detected succeeding 3,4-benzpyrene and in similar concentration. This is reported as having slight activity (Cooper and Lindsey, 1955). Berenblum and Schoental have reported a fraction PES-E eluted im-

FIG. 8.—Fluorescence spectrum of standard 3,4,9,10-dibenzpyrene in cyclohexane.

mediately after benzpyrene which was potent for rabbit and mouse skin and which had fluorescence bands at 412 and 430 m$\mu$, as well as a more strongly adsorbed fraction PES-G which had a visible fluorescence band at 385 m$\mu$, and which was active for rabbit skin. In the present work fractions were obtained which may be identical with both of these fractions.

The potent carcinogen 3,4,8,9-dibenzpyrene was detected in minute amounts. Bonnet and Neukomm (1956) claimed to have detected minute quantities of 3,4,9,10-dibenzpyrene. This compound has a characteristic fluorescence spectrum (Fig. 8). It was not detected in the present investigation.

1,2,7,8-Dibenzfluorene, detected among other members of this class of compounds, has been shown by Badger, Cook, Hewett, Kennaway, Kennaway, Martin and Robinson (1940) to be carcinogenic for mouse skin.

Some of the compounds detected in the present investigation are shown in Fig. 9.

Approximate concentration levels have been ascertained for the benzanthrance derivatives, the 1,2-benzpyrene, 3,4-benzpyrene, 1,12-benzperylene and 3,4,8,9-

dibenzpyrene. They were found in low concentration, e.g. less than 2 p.p.m. of whole tar, although indications are that concentration levels may be stepped up by increasing the frequency of drawing to a rate unrealistic with respect to the human habit. This latter implication, if true, would disqualify as far as aetiological studies of human cancer is concerned, work in which conditions did not approximate to the normal human method of smoking.

Wynder and Wright (1957) showed their basic fraction to have slight though independent carcinogenic activity. It augmented tumour formation by the neutral fraction. This was especially noted for the less susceptible strain of mice ($CAF_1$) in which conditions for assessing promotion phenomena were more likely to have been met.

FIG. 9.—Compounds detected in cigarette smoke tar. (i) 6,7-Cyclopentano-1,2-benzanthracene. (ii) 5,6-Cyclopentano-1,2-benzanthracene. (iii) 11,12-Benzfluoranthene. (iv) 3,4,8,9-Dibenzpyrene. (v) 1,2,3,4,5,6-Tribenzanthracene. (vi) Anthraceno-2′,3′,9,10-phenanthrene. (vii) 1,2,7,8-Dibenzfluorene.

In the light of the biological and chemical investigations to date the following conclusion seems justified : that cigarette tar prepared in a manner approximating to the human smoking method contains sub-threshold levels of various carcinogens, which, acting in concert, and in the presence of a promoting basic fraction, constitute a feebly carcinogenic tar for rodents.

### SUMMARY

An investigation into the composition of a neutral fraction of cigarette tar obtained from British cigarettes in a manner simulating human smoking, was undertaken.

Repetitive adsorption chromatography revealed the presence of compounds, hitherto unrecorded in the present connection, some of which are known to be carcinogenic for rodents. Attention was drawn to certain fractions containing unidentified compounds which may be carcinogenic.

The carcinogenic agents were present in low concentration, e.g. less than 2 p.p.m. whole tar for those estimated, and this fact is discussed in relation to the biological findings to date.

We wish to thank Dr. P. R. Peacock, Director of Research, for helpful advice and for reading the manuscript. One of us (H. J.) is indebted to the Medical Research Council for a grant.

### REFERENCES

Badget, G. M., Cook, J. W., Hewett, C. L., Kennaway, E. L., Kennaway, N. M., Martin, R. H. and Robinson, A. M.—(1940) *Proc. Roy. Soc.*, B, **129**, 439.

Beck, S.—(1954) *Rep. Brit. Emp. Cancer Campgn.*, **32**, 296.

Berenblum, I. and Schoental, R.—(1947) *Brit. J. Cancer*, **1**, 157.

Bergmann, E. D., Fischer, E., Hirschberg, Y., Lavie, D., Sprinzak, Y. and Szmuszkovicz, J.—(1953) *Bull. Soc. chim.*, **20**, 798.

Blacklock, J. W. S.—(1957) *Brit. J. Cancer*, **11**, 181

Bonnet, J. and Neukomm, S.—(1956) *Hel. Chim. Acta*, **205**, 1724.

Chalmers, J. G.—(1954) *Rep. Brit. Emp. Cancer Campgn.*, **32**, 296.

Clar, E.—(1952) ' Aromatische Kohlenwasserstoffe '. Berlin (Springer), p. 205.

Cooper, R. L. and Lindsey, A. J.—(1955) *Brit. J. Cancer*, **9**, 304.

Hamer, D. and Woodhouse, D. L.—(1956) *Ibid.*, **10**, 49.

Hartwell, J. L.—(1951) ' Survey of Compounds which have been tested for Carcinogenic Activity '. Public Health Service, U.S.A., No. 149, p. 237.

Lijinsky, W., Saffiotti, U. and Shubik, P.—(1957) *J. nat. Cancer Inst.*, **18**, 687.

Lyons, M. J.—(1955) *Rep. Brit. Emp. Cancer Campgn.*, **33**, 277.

*Idem.*—(1956) *Nature, Lond.*, **177**, 630.

*Idem* and Johnston, Hilda.—(1957) *Brit. J. Cancer*, **11**, 60.

*Idem*, Peacock, A. and Peacock, P. R.—(1956) *Rep. Brit. Emp. Cancer Campgn.*, **34**, 288.

Passey, R. D., Boyland, E., Pratt, B. M. G. and Hieger, I.—(1956) *Ibid.*, **34**, 15.

Poel, W. E. and Kammer, A. G.—(1957) *J. nat. Cancer Inst.*, **18**, 41.

Sugiura, K.—(1956) *Gann*, **47**, 243.

Wynder, E. L., Graham, E. A. and Croninger, A. B.—(1953) *Cancer Res.*, **13**, 855.

*Iidem.*—(1955) *Ibid.*, **15**, 445.

Wynder, E. L. and Wright, G.—(1956) *Proc. Amer. Ass. Cancer Res.*, **2**, 159.

*Iidem.*—(1957) *Cancer*, **10**, 255.